# RETRACTION: The effect of traditional Chinese medicine soaking method on the healing of diabetic foot ulcers: A meta‐analysis

**DOI:** 10.1111/iwj.70078

**Published:** 2024-09-24

## Abstract

Retraction: HanJ. , ShenJ. , LingB. , “The Effect of Traditional Chinese Medicine Soaking Method on the Healing of Diabetic Foot Ulcers: A Meta‐Analysis,” International Wound Journal 21, no. 3 (2023): e14764, 10.1111/iwj.14764.PMC1091746138447218

The above article, published online on 06 March 2024, in Wiley Online Library (http://onlinelibrary.wiley.com/), has been retracted by agreement between the journal Editor in Chief, Professor Keith Harding; and John Wiley & Sons, Ltd. It came to the publisher's attention from a third party that a number of articles shared concerning similarities in format and structure. Following an investigation by the publisher, the retraction has been agreed on as the peer review and publishing process for this article were found to be manipulated.

Retraction: J. Han , J. Shen , B. Ling , “The Effect of Traditional Chinese Medicine Soaking Method on the Healing of Diabetic Foot Ulcers: A Meta‐Analysis,” International Wound Journal 21, no. 3 (2023): e14764, 10.1111/iwj.14764.PMC1091746138447218

The above article, published online on 06 March 2024, in Wiley Online Library (http://onlinelibrary.wiley.com/), has been retracted by agreement between the journal Editor in Chief, Professor Keith Harding; and John Wiley & Sons, Ltd. It came to the publisher's attention from a third party that a number of articles shared concerning similarities in format and structure. Following an investigation by the publisher, the retraction has been agreed on as the peer review and publishing process for this article were found to be manipulated.

